# Supplementary material for: Use of Oxidative Stress Responses to Determine the Efficacy of Inactivation Treatments on Cryptosporidium Oocysts
Source: Microorganisms. 2021 Jul 8;9(7):1463. doi: 10.3390/microorganisms9071463 (PMC8305202; doi:10.3390/microorganisms9071463)
Supplement: Supplementary file 1 [file microorganisms-09-01463-s001.zip › Supplementary tables and figure Microorganisms.pdf]

List of Supplementary tables and figures

Optimisation of the RNA quality

The quality of RNA was severely affected by the bead-beating condition. Different durations of bead-beating were tested against a fixed speed (4 m/s). It was shown that the quality of RNA was severely affected when the duration of bead-beating was longer than 30 s. The Bioanalyzer results showed that the samples subjected to bead beating of 60 s were of very low quality and the degradation was so severe that no RIN value was reported.

The optimised method included a mild lysis approach with bead-beating only for 25 s and putting the lysate on ice in between the cycles of bead-beating for 3 min.

Table S1. The assessment of quality of RNA, using Agilent's Bioanalyzer, extracted from 10 million oocysts of *C. parvum* under different conditions and kits

Bead-beating condition	RIN	
	RNeasy Plus mini kit	PureLink RNA mini kit
4 m/s for 15 s (2 cycles)	9.2	9.2
4 m/s for 30 s (2 cycles)	9.0	8.5
4 m/s for 60 s (2 cycles)	NA	ND
4 m/s for 20 s (3 cycles)	9.5	ND
4 m/s for 20 s (3 cycles) [a]	NA	NA
6 m/s for 20 s (3 cycles)	NA	NA
6 m/s for 20 s (3 cycles)	7 [b]	ND
6 m/s for 20 s (3 cycles) [a]	NA [b]	ND

[a] garnet beads used instead of the lysing matrix E tube
[b] extracted using QIAcube automated system
ND: not done
NA: RIN not available

Table S2. The overall alignment rate of trimmed reads against the reference genome and transcriptome of *Cryptosporidium parvum*

Sample	Raw reads	Trimmed reads	Overall alignment rate (%)	
			STAR	Salmon
control 1	27878598	26620351	97.96	93.20
control 2	23459177	23075085	97.77	94.90
control 3	17486930	16539962	98.19	94.40
control 4	13061000	12752419	98.40	94.70
Xanth_ox_1	23377041	22510385	96.45	89.30
Xanth_ox_2	19825148	19397343	96.46	89.50
Xanth_ox_3	15911371	15380705	96.40	90.10
Xanth_ox_4	18693182	18319921	97.64	92.30
1M_MSB_1	20636906	19628468	98.75	94.50
1M_MSB_2	24400998	24019388	99.01	95.60
1M_MSB_3	19455385	18888633	98.78	94.70
1M_MSB_4	29212927	28039553	99.03	95.90
0.1M_MSB_1	17954612	16999783	98.68	95.10
0.1M_MSB_2	25264410	24818569	98.70	95.40
0.1M_MSB_3	20265589	19943975	98.61	95.50
0.1M_MSB_4	17021326	16172993	98.42	95.00

Heat_shock_1	25417560	24546200	98.32	92.70
Heat_shock_2	26218711	25825395	98.5	93.70
Heat_shock_3	23328379	22719854	98.42	93.50
Heat_shock_4	21647093	21266741	98.76	94.30

Table S3. List of potential target genes selected for the assessment of the viability of *Cryptosporidium* oocysts using the novel comparative RT-qPCR method

**Gene ID***	**Gene name**	**Log2foldchange**
cgd4_500	COWP7	5.3
cgd8_920	UDP-glucose 6-dehydrogenase	4.8
cgd7_4080	Thioredoxin	4.7
cgd7_4240	Prohibitin	4
cgd7_470	Type 3 Malate dehydrogenase	4
cgd4_3270	HSP70	2.6

* according to https://cryptodb.org/cryptodb/app

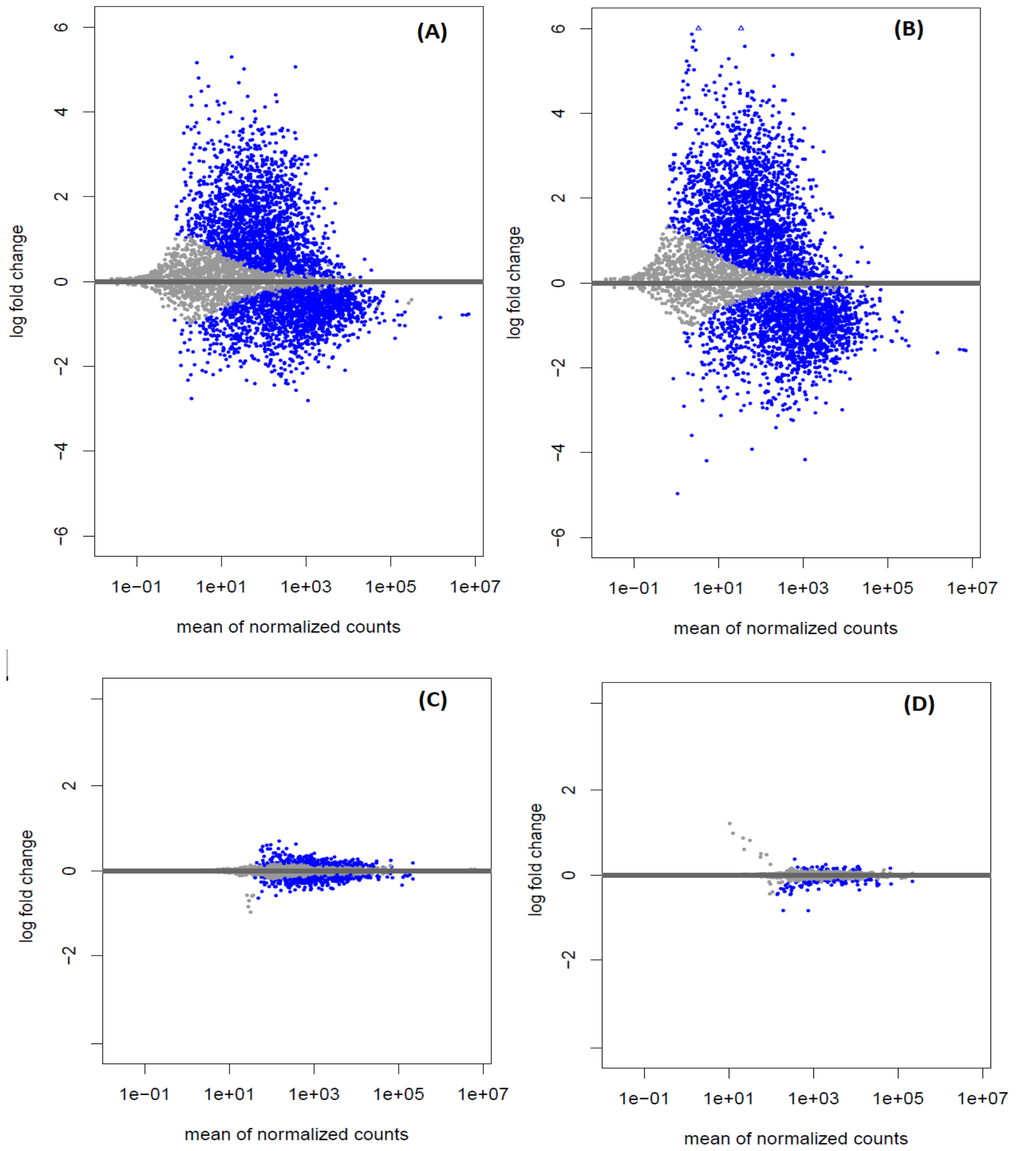

Figure S1. The MA-plot representing gene expression differences following (A) heat shock treatment (37 °C for 20 min), (B) xanthine oxidase and hypoxanthine reaction, (C) 0.1M MSB treatment, (D) 1M MSB treatment compared to the untreated control group.

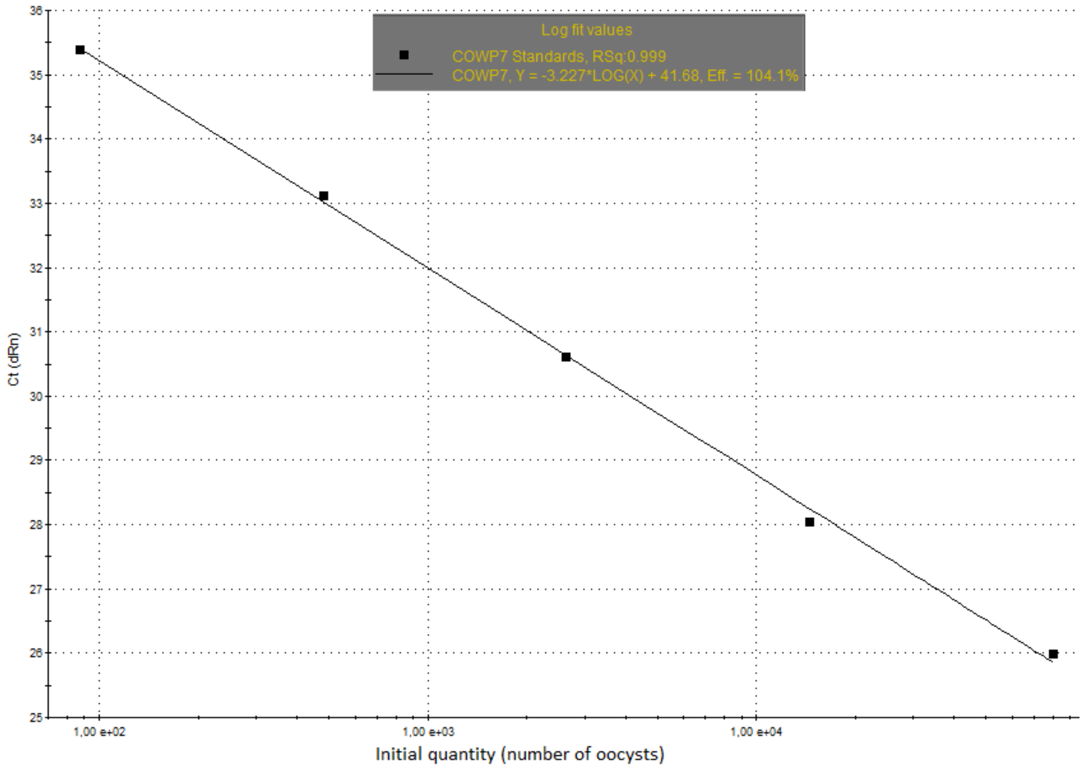

Figure S2. The standard curve prepared by using 5-fold serial dilution of RNA extracted from 2 million oocysts of *C. parvum*.

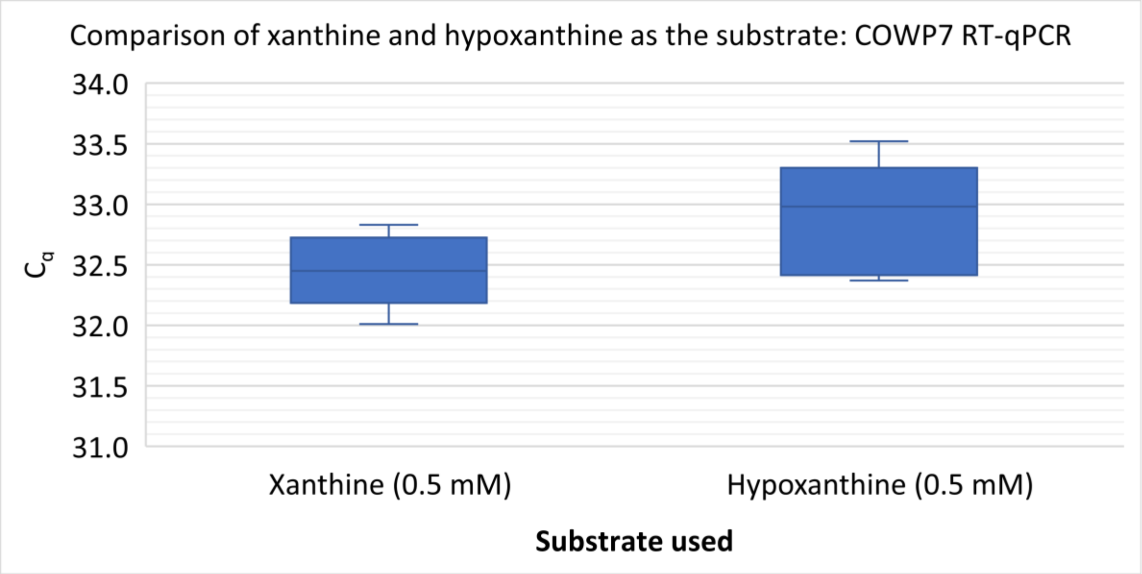

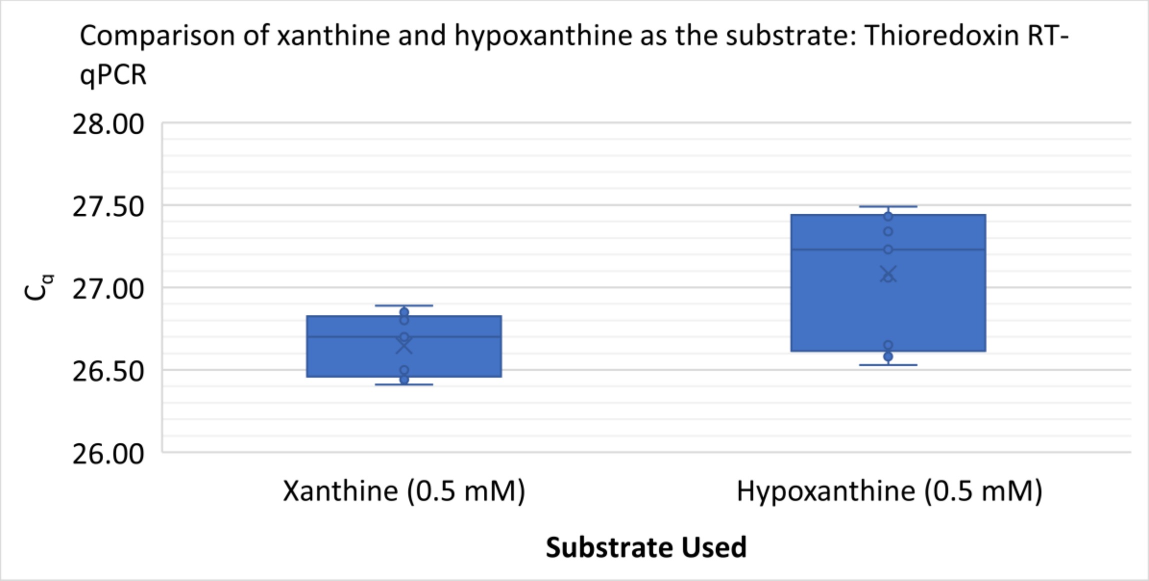

Figure S3. Comparison of xanthine and hypoxanthine as the substrate in the oxidative stress challenge as indicated by RT-qPCR results for COWP7 (top) and thioredoxin (bottom) tests.

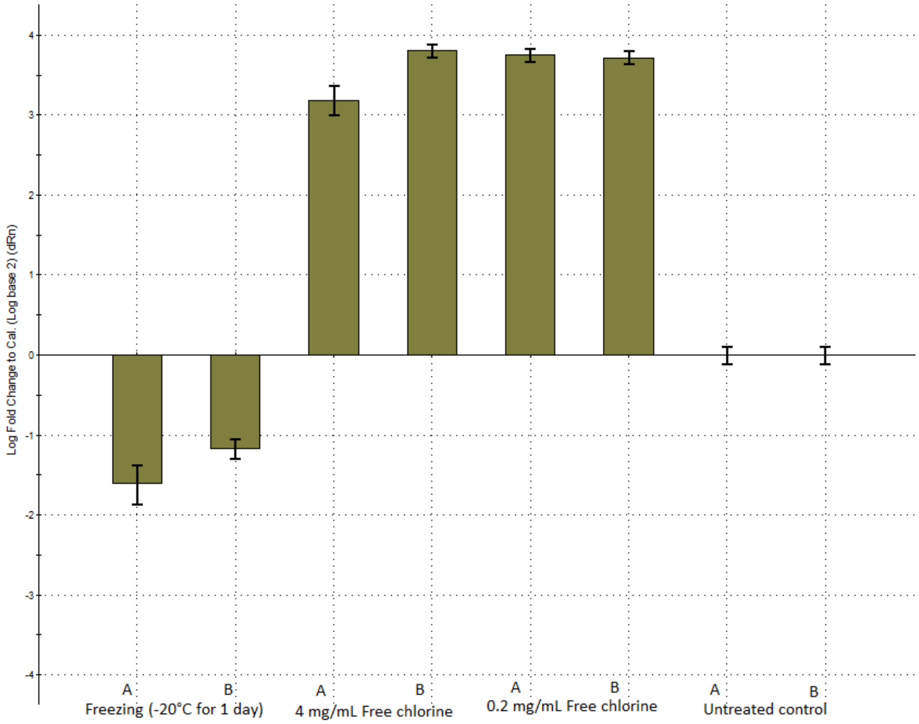

Figure S4. Relative quantity chart for Thioredoxin RT-qPCR test on samples frozen at -20°C for 24 h, treated with 4 mg/L free chlorine, and 0.2 mg/L free chlorine for 30 min (18s rRNA was used as the reference gene).

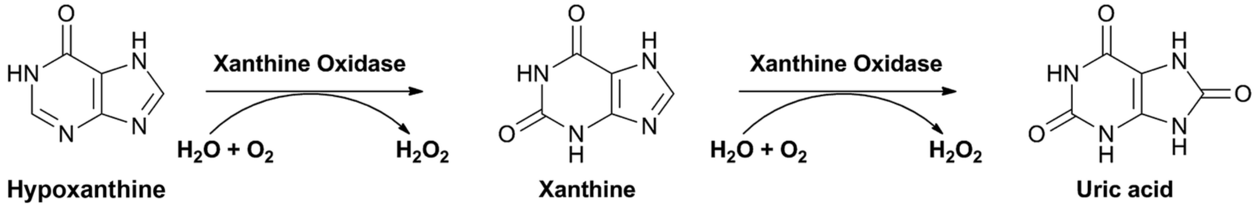

Figure S5. Xanthine oxidase catalyzed reactions. source : [1]

1. Rodrigues, M.V.N.; Corrêa, R.S.; Vanzolini, K.L.; Santos, D.S.; Batista, A.A.; Cass, Q.B. Characterization and screening of tight binding inhibitors of xanthine oxidase: an on-flow assay. *RSC Advances* **2015**, *5*, 37533-37538, doi:10.1039/C5RA01741F.
